# Inpatient screening for social determinants of health: A quality improvement initiative

**DOI:** 10.1017/cts.2024.552

**Published:** 2024-06-03

**Authors:** Dana Kliewer, Brian McGillen

**Affiliations:** Department of Medicine, Penn State Health Milton S. Hershey Medical Center, Hershey, PA, USA

**Keywords:** PRAPARE, readmissions, screening, social determinants of health

## Abstract

**Introduction:**

Social determinants of health (SDOH) can contribute to disparities that negatively impact health outcomes and healthcare utilization. Comprehensive screening is frequently overlooked during inpatient clinical care. This pilot aimed to evaluate the capturability of a multi-domain SDOH screening tool during hospitalization, as well as correlation of SDOH needs to readmissions.

**Methods:**

The Protocol for Responding to and Assessing Patients’ Assets, Risks and Experiences (PRAPARE) screening tool was implemented on admission with adult inpatients at an academic tertiary medical center in central Pennsylvania. A total of 80 patients were screened over an 8-week period using the PRAPARE tool.

**Results:**

43.7% of participants were identified as having at least one SDOH need and 21.2% were identified as having two or more needs. Of the participants identified as having at least one SDOH need through PRAPARE screening, 42.5% experienced a readmission within 30 days, compared to 15% readmissions among participants with no identified SDOH needs. For each additional SDOH need a patient had, the odds they experienced a readmission increased by 2.2 times.

**Conclusions:**

The study findings suggest that utilization of the PRAPARE screening tool has the ability to capture significant SDOH needs among hospitalized patients. This study also suggests that higher SDOH needs correlate to increased odds of experiencing a hospital readmission.

## Introduction

Hospital readmissions represent low-value care within the US healthcare system, with an estimated $26 billion in cost annually attributable to this problem [1,2]. Although current literature suggests that many hospital readmissions are not preventable [3,4], the proportion that are preventable have led to significant penalties enforced on health systems by federal oversight bodies and commercial payers alike. Hospitals and healthcare systems have since gone to great lengths hiring additional care coordination staff and purchasing predictive readmission screening tools to enhance their ability to reduce unnecessary repeat hospitalizations [5].

Many readmission screening tools fail to account for the impacts of social determinants of health (SDOH) – defined as “the conditions in which people live, work, play, and age” [6] – or, at the individual level, health-related social needs (HRSN), on the circumstances that lead to recurrent hospitalizations. There is a plethora of literature that recognizes the negative impact of SDOH/HRSN on health outcomes [7,8] and proposes a direct relationship between disparities and overall healthcare utilization [9–13]. However, the literature surrounding the impact of SDOH on hospital readmissions as a specific segment of healthcare utilization is less clear [14]. The Joint Commission openly states that “understanding of [these needs] is critical for designing practical, patient-centered care plans” [15]. Yet, as opposed to approaches taken in outpatient office-based settings, comprehensive inpatient screening for SDOH/HRSN does not occur as frequently [16]. As a means of improving hospital approaches toward health equity, both the Centers for Medicare and Medicaid Services and the Joint Commission have mandated that SDOH screening be conducted in five mandatory domains [17]. The five SDOH domains include economic stability, education access and quality, healthcare access and quality, neighborhood, and built environment, as well as social and community context [18]. While this represents a move in the direction of improved inpatient SDOH/HRSN screening, it potentially remains suboptimal, as several other domains will remain unaccounted for.

This study focuses on the implementation of the Protocol for Responding to and Assessing Patients’ Assets, Risks and Experiences (PRAPARE) tool, a multi-domain SDOH screening tool, within a pilot period at a single-site academic medical center. The outcome measures sought to examine the capturability of SDOH domains of the PRAPARE tool and any identifiable relationships between SDOH and readmission rates among study participants.

## Material and methods

### PRAPARE tool

The tool implemented was the PRAPARE. The tool itself is a standardized, validated patient risk assessment protocol created by the National Association of Community Health Centers, the Association of Asian Pacific Community Health Organizations, the Oregon Primary Care Association, and the Institute for Alternative Futures [19]. The PRAPARE tool underwent feasibility, usability, and acceptability testing across seven health centers and four states during the pilot study period [20]. During this pilot study, the tool also underwent patient-level data evaluation for internal consistency and reliability [20,21]. The tool contains 21 questions regarding an individual’s personal characteristics, family and home life, and financial and current resources, as well as social and emotional health. The PRAPARE tool has been implemented at various health systems nationally across the care continuum [20,22].

### Setting and participant criteria

Screening was implemented at a large academic tertiary medical center in central Pennsylvania over an 8-week pilot period. The sample of participants was obtained from 44-bed medical-surgical acute care unit. PRAPARE tool screening was implemented for all patients admitted to this unit who met inclusion criteria and consented to participation. The inclusion criteria were (a) inpatient admission under the Internal Medicine service line, (b) anticipated admission for greater than 24 hours, (c) 18 years of age or older, and (d) English speaking. The inclusion criteria of anticipated admission for greater than 24 hours were selected due to the current organizational policy that brief SDOH screening and initial patient assessments by care transitions staff are to be completed within two business days or 48 hours. Patients who experienced shorter hospitalizations may not have completed any baseline screening. The exclusion criteria were (a) observation admission status, (b) inability to adequately interact with project coordinator to complete screening (e.g. Confusion, delirium, intellectual disability), or (c) other special circumstances evaluated on a case-by-case basis (e.g. documented agitation, administration of pharmaceutical restraints/sedatives in previous 24-hour period). The exclusion criteria of observation admission status were selected due to the expected short length of stay for observation patients. Observation status patients also do not receive brief SODH screening or patient assessments by care transitions staff. If after chart review it was unclear to the project coordinator if the patient could adequately participate in PRAPARE screening, the patient’s medical team/provider and bedside nurse were contacted to further assess eligibility.

### Participant recruitment and PRAPARE tool screening

At the beginning of each screening day, a list of the Internal Medicine census was generated and filtered to patients who were admitted within the designated pilot inpatient unit. Each potential participant was prescreened for inclusion or exclusion criteria based on available information within the EHR. Patients who met inclusion criteria were compiled in an electronic screening list. Each potential participant was approached in their hospital room by the project coordinator to provide both a written and verbal overview of the project as well as PRAPARE screening. After this overview was provided, potential participants were then asked to accept or deny PRAPARE screening participation. If agreeable, the PRAPARE screening tool was administered in the patient’s hospital room by the project coordinator. Answers to the PRAPARE tool were hand-written by the project coordinator on a patient-labeled PRAPARE screening tool. A total of 80 patients completed PRAPARE screening over the 8-week period. A total of 245 patients did not meet inclusion criteria or declined participation.

### Retrospective readmission data compilation

Data from completed PRAPARE tools were compiled into a secure spreadsheet. One week after initial PRAPARE screening completion, the EHRs of each of the same sample of participants who completed the PRAPARE tool were accessed to evaluate for any 7-day readmissions from the date of discharge. Thirty days after initial PRAPARE screening completion, the EHRs of each of the same sample of participants were accessed to review for any 30-day readmissions from date of discharge. Periodic chart reviews were also completed if any of the study sample participants experienced prolonged hospitalizations to ensure readmission data was appropriately captured. Following the completion of chart reviews, all identifiable patient information was removed from the secure data spreadsheet and a participant number was assigned to each patient entry.

### Data analysis

To examine the outcome measure of SDOH domain capturability, the number of needs identified was calculated for each domain of the PRAPARE screening tool. To examine the additional outcome measure examining any identifiable relationships between SDOH needs and readmission rates among study participants, a Spearman’s rank correlation coefficient was calculated to assess the strength and direction of relationship between the PRAPARE screening tool and hospital readmissions. The readmission count data was also transitioned from raw numerical data to a dichotomous “yes” or “no” response for each participant to allow for completion of binary logistic regression to assess the impact of SDOH needs on the likelihood of experiencing a readmission. All statistical analyses were completed using Minitab v. 20 web application.

## Results

### Participant characteristics

A total of 80 patients participated in the pilot. Analysis of participant demographics revealed a largely homogenous population with 93.75% of participants identifying as White, 5% identifying as Black/African American, and 1.25% identifying as Alaskan/American Indian. Among this racial profile, 3.75% identified as Hispanic or Latino ethnicity. Demographic data were also obtained from Internal Medicine patient discharges from the same fiscal year as that of the study completion period to allow for comparisons of representation of study participants and overall potential participants. Both sets of demographics are displayed in Table 1.

**Table 1.** Study vs fiscal year (FY) internal medicine discharge patient demographics

Gender	Study demographics	FY* discharge demographics
Male	46 (57.5%)	958 (50.8%)
Female	34 (42.5%)	929 (49.2%)
**Race**		
White	75 (93.75%)	1517 (80.4%)
African American	4 (5%)	127 (6.7%)
Asian	0 (0%)	59 (3.1%)
Declined	0 (0%)	1 (0.1%)
American Indian/Alaskan	1 (1.25%)	179 (9.5%)
Native/Other		
Unavailable	0 (0%)	4 (0.2%)
**Ethnicity**		
Hispanic/Latino	3 (3.75%)	101 (5.4%)
Not Hispanic/Latino	77 (96.25%)	1771 (93.9%)
Declined	0 (0%)	1 (0.1%)
Hispanic origin unknown	0 (0%)	10 (0.5%)
Unavailable	0 (0%)	4 (0.2%)

### Identified SDOH needs

**Figure 1.**
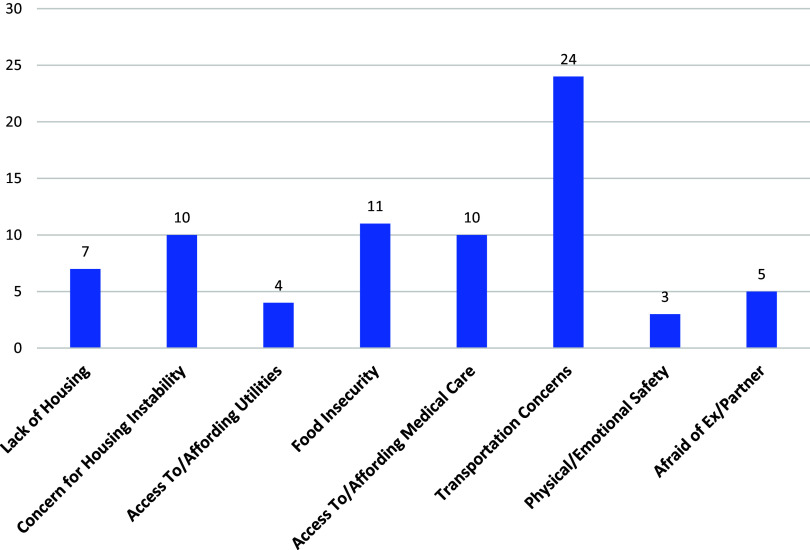
Protocol for Responding to and Assessing Patients’ Assets, Risks and Experiences social determinants of health needs identified.

Of the 80 patients screened, 40 patients had at least one SDOH need identified and 17 patients had two or more needs identified through PRAPARE screening. Twenty-four patients (30%) disclosed that a lack of transportation has kept them from getting to medical appointments, work, or obtaining necessities for daily living. Eleven patients (13.75%) reported food insecurity within the last year. Ten patients (12.5%) disclosed concerns about losing their current housing. Ten patients (12.5%) also noted difficulty with accessing or affording medical care or medications within the last year. Seven patients (8.7%) reported a lack of current housing. Five patients (6.25%) disclosed that they are or were afraid of a partner or an ex-partner within the last year. Four patients (5%) noted an inability to access utilities within the last year. Three patients (3.75%) shared that they did not feel physically or emotionally safe where they are currently living. The PRAPARE tool captured a total of 74 needs. See Figure 1 for SDOH needs identified via PRAPARE screening.

### Readmission data

Retrospective chart reviews revealed that among the 80 participants, there were a total of eight readmissions within 7 days of discharge and a total of 28 readmissions within 30 days of discharge. Five participants had more than one readmission within 30 days of discharge. Of the eight participants who experienced a 7-day readmission, six (75%) were identified as having at least one SDOH need. Among the 40 participants identified as having at least one SDOH need through PRAPARE screening, 17 (42.5%) experienced a readmission within 30 days. Among the 40 participants without SDOH needs identified, six (15%) experienced a readmission within 30 days. A Spearman’s rank correlation coefficient was completed to determine the relationship between the number of SDOH needs and number of hospital readmissions. There was a statistically significant positive correlation between the two variables (*r*_s_ = 0.425, *p* < 0.001).

**Figure 2.**
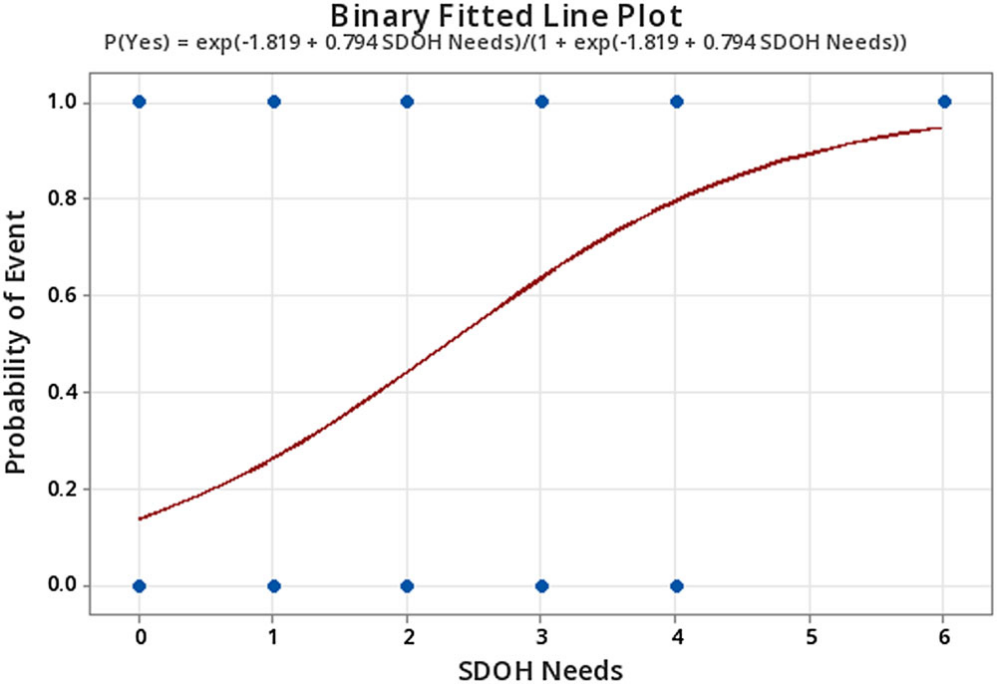
Binary fitted line plot examining social determinants of health needs and probability of 30-day hospital readmission.

Binary logistic regression output revealed a coefficient for SDOH needs of 0.76, with an associated *Z*-value = 3.49, *p* < 0.001. These results suggest that the effect of SDOH needs on the likelihood of experiencing a 30-day hospital readmission is statistically significant. Analysis further yielded an odds ratio of 2.21 for SDOH needs with a 95% confidence interval [1.41, 3.45]. To interpret this result, for each additional SDOH need a patient has, the odds that they experience a readmission increase by approximately 2.2 times. See Figure 2 for the binary fitted line plot examining SDOH needs and probability of 30-day readmission.

## Discussion

### Limitations

This pilot was limited by the selection of a population of English-speaking participants. Analysis of pilot demographics revealed a largely White and non-Hispanic sample with notably higher percentages of representation when compared to the fiscal year discharge demographic data in Table 1. This homogenous sample may have inadvertently biased results by not capturing data from those within minority ethnic or racial populations, which have historically been recognized in the literature to have higher health disparities nationally [23,24]. It is also important to note that the catchment area of the study site does include a large rural area, which may have contributed to the similar disparity types identified.

Although each study participant answered every question within the PRAPARE tool and there were no missing data points, there may have been potential issues with non-disclosure. Disclosures of social needs may vary depending on the context and constraints under which the screening tool was administered. Greater disclosure may have been elicited with different screening approaches, such as paper self-administration or electronic completion rather than via project coordinator interview.

### Results and future implications

Overall, the study findings suggest that utilization of the PRAPARE screening tool has the ability to capture significant SDOH needs among hospitalized patients. This study also suggests that higher SDOH needs correlate to increased odds of experiencing a hospital readmission. Future research should focus on administration of PRAPARE screening universally to hospitalized patients to better identify those at greatest risk, which could yield important health benefits for those affected and overall cost savings for the health system.

At present, the pilot findings were impactful at the microsystem level. The overall project results are consistent with those found within the literature and support ongoing multi-domain screening evidence in practice. There is future potential to improve the overall patient-care-continuum and health outcomes at the macrosystem level through application of these results, engagement of stakeholders, and system-wide adoption of standardized multi-domain screening practices.

The findings from this pilot also highlight the need to identify a more sustainable method for PRAPARE screening and streamline results into the electronic health record for ease of access among care team members. Ideally, the PRAPARE tool would be completed upon admission for all hospitalized patients. Health systems could also explore options for screening tool self-administration for patients who are able to reduce the overall burden on direct-care staff. Hospital-wide PRAPARE screening would require staff buy-in, screening tool education, and consideration if clinical-decision support or hard stops should be built into the interfacing to improve completion rates. Future organizational initiatives should focus on acceptance and use of a standardized, validated multi-domain tool across the health system, integration of the multi-domain tool into the EHR, and generating a database of resources for unmet SDOH needs.
